# Poorly Differentiated and Anaplastic Thyroid Cancer: Insights into Genomics, Microenvironment and New Drugs

**DOI:** 10.3390/cancers13133200

**Published:** 2021-06-26

**Authors:** Alessandro Prete, Antonio Matrone, Carla Gambale, Liborio Torregrossa, Elisa Minaldi, Cristina Romei, Raffaele Ciampi, Eleonora Molinaro, Rossella Elisei

**Affiliations:** 1 Endocrine Unit, Department of Clinical and Experimental Medicine, University Hospital of Pisa, Via Paradisa 2, 56124 Pisa, Italy or alessandro.prete22@gmail.com (A.P.); antonio.matrone@med.unipi.it (A.M.); gambalecarla@libero.it (C.G.); elisa.minaldi@med.unipi.it (E.M.); cristina.romei@unipi.it (C.R.); raffaele.ciampi@unipi.it (R.C.); e.molinaro@ao-pisa.toscana.it (E.M.); 2 Department of Surgical, Medical, Molecular Pathology and Critical Area, University Hospital of Pisa, 56124 Pisa, Italy; l.torregrossa@ao-pisa.toscana.it

**Keywords:** anaplastic thyroid cancer, poorly thyroid cancer, genetic landscape, tumor microenvironment, genetically guided therapy

## Abstract

**Simple Summary:**

In the last decades, many researchers produced promising data concerning genetics and tumor microenvironment of poorly differentiated thyroid cancer (PDTC) and anaplastic thyroid cancer (ATC). They are trying to tear the veil covering these orphan cancers, suggesting new therapeutic weapons as single or combined therapies.

**Abstract:**

PDTC and ATC present median overall survival of 6 years and 6 months, respectively. In spite of their rarity, patients with PDTC and ATC represent a significant clinical problem, because of their poor survival and the substantial inefficacy of classical therapies. We reviewed the newest findings about genetic features of PDTC and ATC, from mutations occurring in DNA to alterations in RNA. Therefore, we describe their tumor microenvironments (both immune and not-immune) and the interactions between tumor and neighboring cells. Finally, we recapitulate how this upcoming evidence are changing the treatment of PDTC and ATC.

## 1. Introduction

Thyroid cancer is the most common endocrine tumor and its incidence has been raising up over the last 20 years, mostly due to the flowering diagnosis of micro thyroid carcinomas [1]. Thyroid cancer is subcategorized into follicular and non-follicular derived carcinoma (e.g., medullary thyroid carcinoma). Among the first, World Health Organization (WHO) identifies papillary thyroid carcinoma (PTC), follicular thyroid carcinoma (FTC), poorly differentiated thyroid carcinoma (PDTC), and anaplastic thyroid carcinoma (ATC) [2].

**Figure 1:**
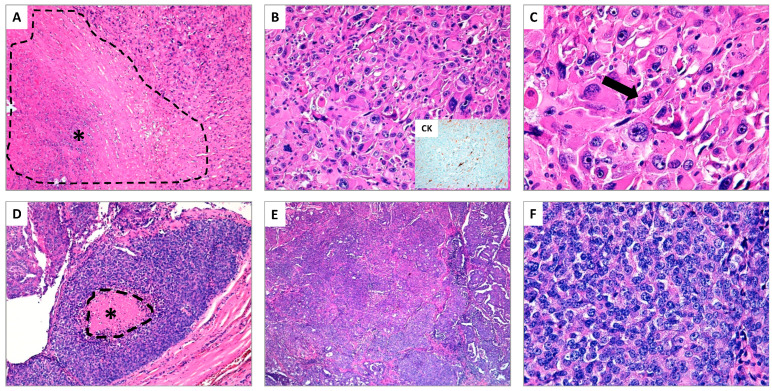
(**A**–**C**) Anaplastic thyroid carcinoma (ATC) (hematoxylin and eosin stain); (**A**) The presence of extensive tumor necrosis is a typical aspect of ATC (see asterisk *) (original magnification ×10); (**B**) The neoplastic cells show marked nuclear atypia with spindled and pleomorphic morphology, associated to elevated mitotic rate, simulating high-grade pleomorphic sarcoma. In the insert, focal immunostaining for cytokeratins supports the epithelial nature of ATC (original magnification ×20); (**C**) At higher magnification, the pronounced nuclear atypia and an atypical mitosis (see arrow) are shown (original magnification ×40). (**D**–**F**) Poorly differentiated thyroid carcinoma (PDTC) (hematoxylin and eosin stain); (**D**) The typical example of PDTC is the so-called “insular carcinoma”. In this field, the tumor shows a small focus of tumor necrosis (see asterisk *) (original magnification ×10); (**E**) The neoplasm exhibits a prevalent solid growth pattern (original magnification ×20); (**F**) At higher magnification, the tumor cells appear small and uniform, the nuclei are generally rounded and hyperchromatic, in absence of the typical aspects of papillary thyroid carcinoma (original magnification ×40).

Thyroid cancer 5 year-survival is variable among the different hystotypes. According to a recent epidemiological study performed in Denmark by using a national cancer registry, the 5 year-survival rates were 91.1% and 79.9% in PTC and FTC, respectively, 63.6% in PDTC and 12.2% in ATC [3]. Unfortunately, PDTC and ATC median overall survival is 6 years and 6 months, respectively [4,5]. Although PDTC and ATC are rare, therapy for patients affected by PDTC and ATC represents an unmet clinical need that should be addressed, considering their poor survival. In addition, PDTC and ATC harbor diagnostic pitfalls that make difficult their clinical management. Although PDTC was added in WHO classification in 2004, its diagnostic criteria are not widely shared and many pathologists are following criteria of Turin consensus conference [6] and others Memorial Sloan Kettering Cancer Center ones [7]. Likewise, the wide spectrum of ATC hystotypes could challenge the differential diagnosis with other cancers (e.g., angiomatoid variant ATC with thyroid angiosarcoma) [8] or even with benign lesions (e.g., acute thyroiditis) [9] (Figure 1).

In the past, many treatments were proposed to answer this aforementioned need, but with disappointing results [10]. Nowadays many clinicians are proposing genetically guided treatments for PDTC and ATC, according to the new discoveries about their genetic landscape [11,12].

In the current review, as first, we summarize the upcoming findings about genetic features of PDTC and ATC, from mutations occurring in DNA to alterations in RNA; therefore, we describe their tumor microenvironments and the interactions between tumor and other neighboring cells; finally, we recapitulate how this upcoming evidence are changing the treatment of PDTC and ATC.

## 2. Genetics Features

Genomic instability is universally considered as a driver of carcinogenesis, supporting the generation of all hallmarks of cancer (i.e., resistance to cell death, promotion of proliferative signaling, escape from growth suppressors, invasion and metastasis capacity, activation of replicative immortality, evasion of immune destruction, deregulation cellular energetics and neo-angiogenesis) [13,14]. Across several neoplasia, thyroid cancer presents lower genomic instability, expressed as the number of mutations per tumor, compared to other adult neoplasia (e.g., endometrial and colorectal cancers) [15]; this evidence has also been confirmed in metastatic cases [16]. However, thyroid cancer shows a heterogenous mutational burden across its histotypes: ATC presents an increased number of genetic alterations per tumor (median 4, range 0–29) compared to PTC and FTC [17]; likewise, according to data from Tissue Cancer Genome Atlas (TCGA), PDTC mutational burden is higher than PTC, even if lower than ATC [18,19]. Genomic instability in PDTC and ATC embraces both somatic driver mutations and gene fusions.

### 2.1. Somatic Driver Mutations

Vogelstein et al. considered a driver mutation as a genomic variant that directly or indirectly induces a selective growth advantage [20]. As shown in Table 1, ATC and PDTC harbor many driver mutations, occurring mainly in both MAPK and PI3K-AKT pathways.

BRAF and RAS genes (HRAS, KRAS, and NRAS) are main members of MAPK pathway. Both of them occur in more than 25% of ATCs, according to catalogue of somatic mutations in cancer (COSMIC) database [21], while 15.38–33.33% and 6.8–41.2% of PDTCs harbor BRAF and RAS mutations, respectively (Table 1) [18,22,23,24]. Interestingly, although BRAF and RAS mutations are present in a relevant percentage of both ATC and PDTC cases, they seem to play different roles. In ATC, neither BRAF or RAS mutations seem to be sufficient to induce neoplastic cell anaplasia. McFadden et al. produced a thyroid-specific CreER transgenic mouse in order to specifically induce BRAF^V600E^ mutation in thyroid cells; although this mutation induces PTC foci, it was capable to promote ATC tumorigenesis only in the presence of p53 mutation [25]. Likewise, KRAS^G12D^ mutation developed anaplastic foci with complete deregulation of normal thyroid follicular morphology in mice model only in association with a homozygous mutation of TSH receptor [26]. However, BRAF-RAS signaling retains a crucial role in ATC cells and its inhibition by siRNA anti-BRAF produces growth arrest in ATC cell lines [27], even stronger in combination with MEK inhibition [25]. Otherwise, the mechanisms seem to be different in PDTC: Vitagliano et al. were able to promote progression of FTC foci into PDTC in mouse model by NRAS^G61K^ mutation [28].

In addition to mutations of MAPK pathway, next generation analysis showed that ATC harbors higher prevalence of mutations in PI3K-AKT pathway compared to other histotypes [30]: according to COSMIC database, PI3KCA and PTEN were found mutated in 11.24% and 9.27%, respectively (Table 1) [21]. Likewise, also PDTC harbors frequently PIK3CA or AKT1 mutations (2.38–19.51% and 0–8.70%, respectively) (Table 1) [18,22,23,24].

Interestingly, in ATC series provided by Liu et al., the 81.3% of samples presented genetic alterations affecting both MAPK and PI3K-AKT pathways [31]. Accordingly, in mouse model, the presence of mutations occurring in both pathways induced ATC foci, confirming the synergistic interactions between these pathways [32]. On one hand, MAPK pathway has a crucial role in cell proliferation and survival, and, on the other hand, upregulated PI3K-AKT pathway has been related to tumor aggressiveness [33].

Beyond mutations occurring at members of MAPK and PI3K-AKT pathways, many variants have been reported in cell cycle regulators. Many reports showed that mutations occurring in p53 and TERT promoter (pTERT) are highly prevalent in ATC, occurring even simultaneously [17,18,21] (Table 1). Likewise, PDTC presents both mutations, even if less frequently than ATC [18,34]. Intriguingly, in the presence of an impaired cell-cycle checkpoint pathway (e.g., p53), the occurrence of a concomitant mutation in telomerase activity (e.g., pTERT) could induce an indefinite cell proliferation [35]. In addition, the interplays between the duet BRAF-pTERT have recently been described by Tan and colleagues [36]. In particular, in case of mutation of both of them, cancer cells suppress apoptosis mainly thank to pTERT activity, while in case of mutation occurring only on BRAF gene, apoptosis activity seems to be not significantly affected [36]. Accordingly, the inhibition of TERT activity could represent an Achilles heel, as recently shown in-vitro and in-vivo model by Bu et al. In these models, BIBR1532 (a TERT inhibitor) significantly inhibited tumor growth as well as cell invasion, migration and angiogenesis [37].

If regulation of cell cycle has a crucial role in oncogenesis, also protein metabolism control has been deeply involved in tumorigenesis [14,38]. Not surprisingly, both PDTC and ATC harbor EIF1AX mutations in about 10% of cases (Table 1) [18,22,23,24]. EIF1AX is a member of 43S preinitiation complexes, responsible of translation initiation, and its mutation has recently been involved in preinitiation complex stabilization and, further, in deregulating protein synthesis [39,40]. Interestingly, EIF1AX mutations are mutually exclusive with other drivers in PTC [19], while they co-occur with RAS mutations in ATC and PDTC [18]. Recently, Krishnamoorthy et al. showed a positive feedback relationship between RAS and EIF1AX proteins, which reinforces c-MYC gene expression [40].

### 2.2. Gene Fusions

Fusion genes are common driver mutations described in both hematopoietic and solid tumors [41]. They usually involve a driver gene, which expresses a receptor tyrosine kinase (e.g., RET) or its downstream kinase (e.g., BRAF), and a partner gene (e.g., NCOA4). If in physiologic state most of these kinases require the ligand to induce their dimerization, these rearrangements are capable to induce a ligand-independent dimerization and a deregulated kinase activity [42]. In the past, all the tumorigenic effects were considered as consequence of a non-controlled expression of the driver gene; however, new evidence suggests that also the partner gene may play a crucial oncogenic role [43].

Although fusion genes have been extensively described in thyroid cancer, their prevalence is lower compared to other solid tumors [41]. PDTC harbors gene fusions in 10–14% of cases while ATC in 3–5% [44] (Table 2). Interestingly, when present, fusions usually involve the same few oncogenes. RET fusions are the most common, mainly CCDC6-RET (RET/PTC1) and NCOA4-RET (RET/PTC3), while NTRK, ALK and BRAF fusions are quite rare (Table 2) [44]. Recently, Nikitski et al. developed a mouse model of STRN-ALK fusion gene that was capable of inducing PTC, PDTC and ATC foci [45]. This model revealed the presence of two clusters of PDTC with specific cell morphology, immunohistochemical characteristics and different levels of expression of thyroid differentiation markers [45].

Although rare, gene fusions could represent precious targets for targeted therapies. Moreover, any histotype of thyroid cancers with gene fusions has recently been proposed as a discrete group with specific histologic characteristics such as multinodular growth and extensive fibrotic features. For this reason, they have been named “kinase fusion-related thyroid carcinomas” [46].

### 2.3. Copy Number Variations

In oncology, copy number variations (CNVs) are well characterized as prognostic factors for recurrence and death [47]. This evidence has been confirmed also in advanced thyroid cancer [18]. If they are quite rare in differentiated thyroid cancer (less than 10%) [19], in PDTC and ATC they are widespread, especially in cancers without known driver mutation (losses of 1p, 8p, 13q, 15q, 17p, 22q, and gains of 1q and 20q) [18]. Interestingly, they seem to be hystotipes-specific: 8p and 17p losses and 20q gains are more frequent in ATC while loss of 1p was substantially more recurring in PDTCs [18]. Moreover, CNVs correlate with gene context where occur: 1p, 13q, and 15q losses were enriched in PDTCs without known driver mutation while loss of 22q was associated with RAS-mutated PDTCs [18]. In ATC, beyond large chromosomal variations, Pozdeyev et al. reported more restricted CNVs such as losses of CDKN2A and CDKN2B or amplification of KIT, PDGFRA and KDR, further confirmed by other authors [17,24].

Finally, since CNVs have recently been related to resistance to target therapies in thyroid cancer [48], it would be very interesting to ascertain if some of them (e.g., PDGFRA amplification) could induce resistance to target therapy (e.g., multikinase inhibitors, MKIs) in ATC and PDTC.

### 2.4. RNA Alterations

It is universally recognized that messenger RNA (mRNA) synthesis and translation are deeply modified in cancer; however, new evidence shows that all kinds of RNA are universally impaired [49]. In normal condition, cells produce different types of RNAs: mRNA, ribosomal RNA (rRNA), transfer RNA (tRNA), microRNA (miRNA), long non-coding (lncRNA) and circular RNA (circRNA). Accordingly, neoplastic cells could deregulate all kinds of RNAs that could promote the cells growth and invasiveness [49].

**Figure 2:**
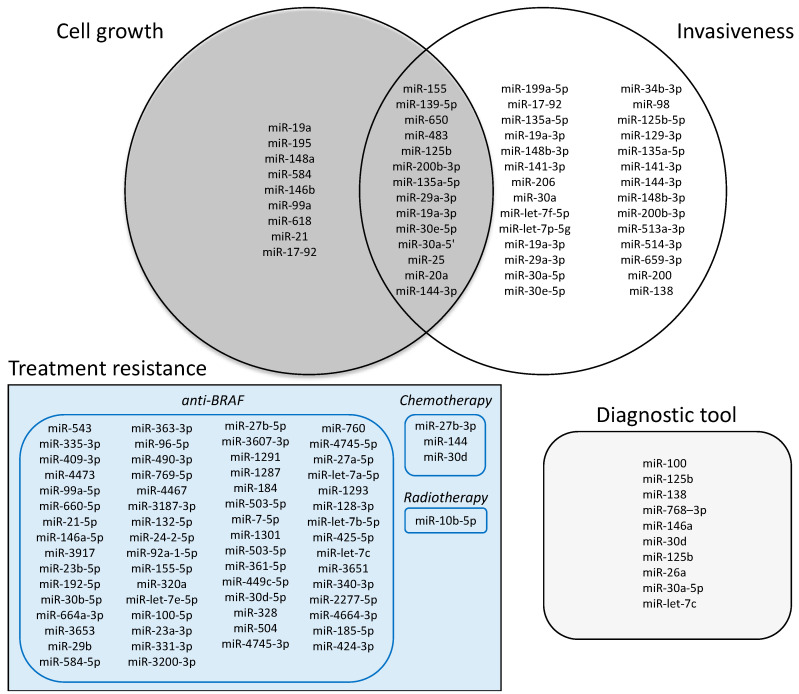
miRNA that were discovered in ATC according to their function.

In particular, miRNA are usually 20–23 nucleotides in length that can bind multiple mRNA, regulating their catabolism and further their translation [50]. 127 and 18 different miRNAs have been characterized in ATC and PDTC, respectively. Among them, 69 miRNAs resulted decreased and 54 increased in ATC, while 10 resulted decreased and 8 increased in PDTC. If their role in PDTC is not fully elucidated and they might be used as an ancillary diagnostic tool and prognostic marker [51], they have been fully characterized in ATC. According to literature data, we grouped them into 3 main roles: regulation of growth tumor, invasiveness and resistance to therapy (Figure 2). We found that 9 miRNA were related to tumor growth [52,53,54,55,56,57,58,59,60,61], 14 to tumor growth and invasiveness [62,63,64,65,66,67,68,69], 28 to invasiveness [67,70,71,72,73,74,75,76], and 66 to therapies resistance (62 to anti-BRAF treatment, 3 to chemotherapy and 1 to radiotherapy) [77,78,79,80,81]. Additionally, 10 miRNAs were considered as an ancillary diagnostic tool [82,83] (Figure 2).

Beyond miRNA, growing evidence is showing the role of lncRNAs in cancer. lncRNA are RNAs longer than 200 nucleotides that do not encode proteins but regulate gene expression, splicing and nucleation of subnuclear domains [49]; moreover, lncRNAs may have cytoplasmic functions, such as miRNA sponging, interaction with signaling proteins, and further modulation of mRNA translation [49]. In ATC, lncRNAs may regulate tumor growth [84,85], invasiveness [86], and both tumor growth and invasiveness [87,88,89,90,91]; they can also regulate cancer sensitivity to treatments [92]. In particular, lncRNA PTCSC3 was described at low levels both in ATC tissue and cell lines and it was demonstrated that its upregulation inhibited the resistance to doxorubicin by suppressing stem cell proprieties [92].

Finally, circRNAs are usually consequence of back-splicing events, producing in a covalently closed circRNA molecule instead of linear ones. Although circRNAs have usually been detected at low levels in normal and in cancer cells, some of them are at higher concentration and have functional roles: miRNA sponging and proteins stabilization [49]. In ATC, a recent study showed that circRNA may produce resistance to chemotherapy. In particular, Liu et al. showed that circRNA EIF6 could sponge miR-144-3p to promote autophagy and cisplatin-resistance [93].

## 3. Tumor Microenvironment

Tumor microenvironment (TME) is the dynamic milieu that harbors tumor cells [94]. It comprises blood vessels, extracellular matrix (ECM), non-neoplastic cells, and signaling molecules [95]. Neoplastic cells interact with other TME members in order to regulate self-growth, invasiveness and resistance to therapy [94]. In thyroid, many reports showed that TME may promote tumor growth, metastatic power, and resistance to therapy, both in differentiated and anaplastic thyroid cancer [96,97,98].

In TME we should distinguish not immune and immune related cells. Among the former, cancer associated fibroblasts play a relevant role in both ATC and PDTC [99,100]. In ATC, tumor cells present paracrine communication with fibroblast: ATC cells activate fibroblasts by reprogramming their metabolism, phenotype and secretome, and then activated fibroblasts reinforce thyroid cancer progression, by enhancing tumor invasion and proliferation [100]. Likewise, interactions between PDTC cells and cancer associated fibroblasts may potentiate tumor progression, by collagen remodeling [99]. Analog interplays have been recently demonstrated between ATC and endothelial cells, partially rescued by sorafenib [94].

Giannini et al. provided significant evidence about immune TME in ATC and PDTC [101]. ATC TME was enriched of tumor infiltrating leukocytes (both macrophage and lymphocytes) and characterized by hot or altered–immunosuppressed phenotype, since a relevant part of CD8+ lymphocytes presented exhausted features. Accordingly, Caillou et al. showed that tumor-associated macrophages build up a dense network in whom cancer cells reside [102,103] and their presence is associated with a worse prognosis in ATC [104]. Cameselle-García and colleagues elucidated that ATC tumors are enriched of tumor infiltrating lymphocytes (mainly CD8+ cytotoxic T cells), which mainly reside in the interface between tumor ant thyroid tissue [105]. Otherwise, PDTC harbored less tumor infiltrating leukocytes compared to ATC, and presented a cold immune contexture in 65% of cases [101]. In these immune contexts, PD/PD-L1 pathway (programmed cell death protein-1/programmed cell death ligand-1) plays a crucial role in ATC and less frequently also in PDTC [97,105,106]. If in physiologic conditions, PD/PD-L1 pathway regulates T cell immune suppression, in neoplastic milieu it is exploited by cancer cells in order to avoid immune attack, by inducing T-cell exhaustion [107]. In ATC, PD/PD-L1 proteins expression was shown to be regulated by BRAF mutation and is was associated to a worst prognosis [106,108]. Accordingly, Brauner et al. demonstrated that dual inhibition of BRAF and PD/PD-L1 pathways induced a powerful shrinkage of ATC tumor in orthotopic immune-competent mouse model [108].

## 4. Contemporary Treatment in ATC and PDTC

**Figure 3:**
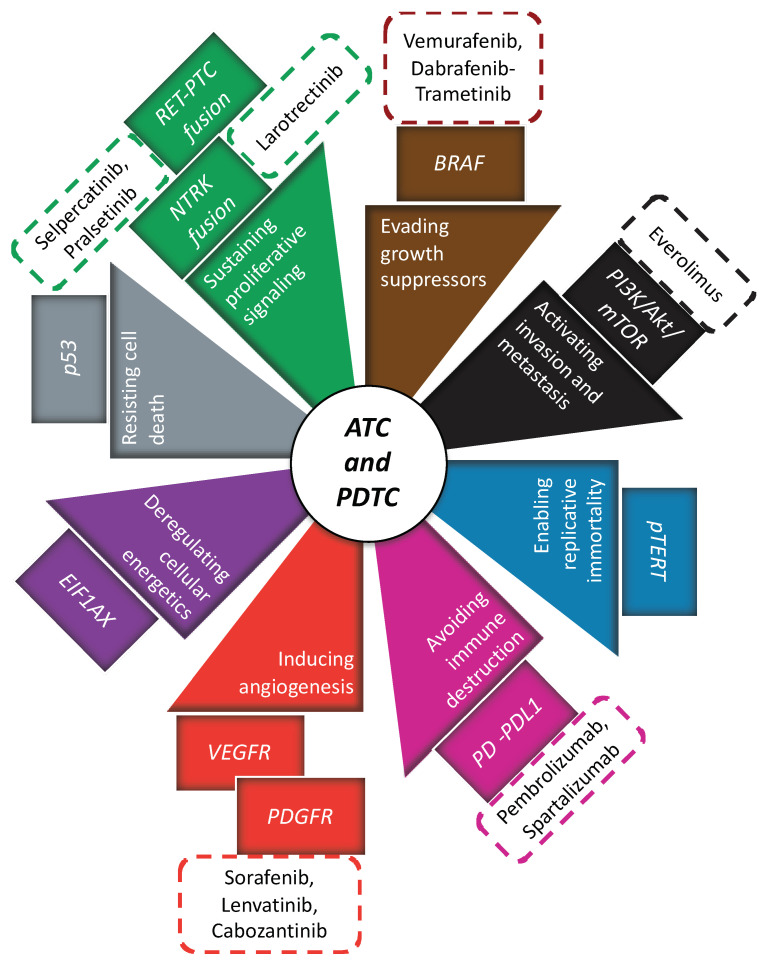
All innovative therapies that have been suggested in ATC and PDTC (rectangular box with dotted lines); each of them has been related to a specific inhibition of hallmark of cancer (triangle box) and cellular pathway (rectangular box).

Traditionally, treatments against ATC and PDTC globally provided disappointing results [12,109]. ATC presents very low median overall survival [109,110]. Although PDTC presents higher 5-year survival (62%) compared to ATC [111], disease control in patients with metastatic PDTC is still poor (59%) and 85% of their disease specific deaths is related to the presence of distant metastasis [12]. In both of them, surgery represents a cornerstone of multimodal treatment; nonetheless, systemic treatment is necessary in case of diffuse disease. Systemic treatment comprises chemotherapy (in case of ATC, elsewhere reviewed [112]), anti-angiogenic therapy, immunotherapy and genetically guided therapy (Figure 3). Since their individual use provided encouraging but insufficient results, they have recently been proposed in multimodal approach.

### 4.1. Antiangiogenic Therapy

As previously shown, neo-angiogenesis is a main hallmark of cancer, sustaining its limitless growth [113]. Accordingly, neoplastic cells regulate neo-angiogenesis in order to guarantee their progression in ATC as well as in PDTC [114,115]. Many anti-angiogenic drugs have been employed to inhibit ATC and PDTC growth. Year by year, sorafenib, lenvatinib, cabozantinib, pazopanib, gefitinib and imatinib have been used with fluctuating results.

Sorafenib was the first anti-angiogenic drug proposed for ATC treatment. It was employed in two different phase-2 trials [116,117]; in both of them, disease control (partial response and stable disease) was reached in about 40% of patients but the median overall survival was still lower than 5 months [116,117]. Similarly, sorafenib seemed not to produce exciting results in PDTC [118]. From 2009 to 2011, 40 patients with PDTC were randomly allocated to sorafenib and placebo arms in a randomized, double-blind, multicentric, phase-3 trial (DECISION trial). Although not statistically significant, the sub analysis on PDTC showed a consistent improvement of PFS in patients treated with sorafenib as in all the other histotypes [119]. As reported in DECISION trial, sorafenib toxicity is mainly characterized by grade 1 and grade 2 adverse events such as hand–foot skin reaction, diarrhoea, alopecia, rash/desquamation, fatigue, weight loss, and hypertension [119].

Lenvatinib had very promising results in in-vitro and in-vivo models of ATC that were partially confirmed in clinical settings [120]. Takahashi et al. performed a phase II clinical trial, showing an overall survival of 10.6 months (95% CI: 3.8–19.8) and disease control in 16 out of 17 patients [121]. However, different results were given by a recent post-marketing observational study which reviewed 124 patients affected by ATC and treated with lenvatinib [122]. It showed a disease control in 76.2% (66.89–83.96%) of patients; however, this response seemed to be only transient, because the time-to-treatment failure was 74.5 (57.0–108.0) days, and the median overall survival was still poor (3.4, 95% CI 2.66–4.33 months) [122]. At the same time, baseline clinical conditions of enrolled patients were poor (ECOG > 1) in more than 70% and this could partially explain these disappointing findings. Further studies should be employed in order to verify lenvatinib efficacy in ATC patients with better clinical conditions. Otherwise, lenvatinib produced interesting results for PDTC patients. In SELECT trial, which explored lenvatinib efficacy in patients with radioiodine-refractory thyroid cancer, 28 patients harboring PDTC were enrolled. In this selected population, lenvatinib confirmed its efficacy compared to placebo (HR 0.21, 0.08–0.56) [123]. Accordingly, a retrospective multicentric analysis of real-world data confirmed these encouraging results [124]. In this analysis of clinical practice in Austria enrolling 43 patients, the overall survival seemed to be not modified by tumor subtype (differentiated vs. poorly differentiated/anaplastic TC), whereas a maintenance dosage higher than 14 mg was associated with better prognosis [124]. About toxicity, in spite of high proportion of adverse events with grade ≥ III in SELECT trial (75.9%), Austrian, Italian, and French real-world data reported lower rates of adverse events of grade ≥ III (44%, 22.3% and 48%, respectively) [123,124,125,126]. Fatigue, hypertension, diarrhea, decreased weight, stomatitis, and anorexia are the most common reported adverse events [125,126].

More recently, cabozantinib has been explored as a salvage therapy for patients with radioiodine-refractory thyroid cancer already treated with MKIs. In this trial, 7 patients with PDTC were enrolled to receive cabozantinib and all of them presented clinical benefit (3 PR and 4 SD) [127]. Other antiangiogenic agents (pazopanib, gefitinib and imatinib) were employed for ATC therapy but they did not produce encouraging results [128,129,130].

### 4.2. Genetically Guided Therapy

Many reports showed that ATC has a singular genomic and transcriptomic landscape (ATC-like) [131]. In this singular genomic landscape, BRAF-MEK pathway was proposed as a potential target.

After the exciting results of BRAF inhibition in BRAF-mutated melanoma [132], a multicenter prospective “basket” trial, encompassing tumors with BRAF mutation, enrolled 7 patients affected by ATC for treatment with vemurafenib: 2 of them experienced a durable partial response (more than 11 months) [133]. In order to produce a stronger inhibition of BRAF-MEK pathway, dual inhibition of BRAF and MEK with dabrafenib and trametinib was proposed. Accordingly, in in-vitro ATC model, combined therapy induced greater growth inhibition than single agents [134]. Likewise, dabrafenib-trametinib therapy produced about 80% of 12-months progression free survival and overall survival in phase II clinical trial enrolling 16 patients [135]. Moreover, 1 patient experienced a complete response, 10 partial response, 3 stable disease and only 1 disease progression. In this trial, fatigue (44%), pyrexia (31%), and nausea (31%), were the most common adverse events, although the 50% of enrolled patients reported an adverse event with grade ≥ III [135]. This trial permitted the approval of this combination by FDA for treatment of BRAF^V600E^ mutated ATC.

As previously reported, PI3K/Akt/mTOR has a crucial role in ATC cells and also anti-mTOR inhibitors have been proposed with conflicting results. Everolimus was used to treat one patient with ATC harboring a mutation of Tuberous Sclerosis 2 protein (TSC2), member of PI3K/AKT/mTOR pathway, obtaining an extraordinary 18-month response [136]. However, these promising results were not confirmed in other studies [137,138].

New perspectives have recently been opened for patients with ATC or PDTC harboring RET fusion genes, since highly selective RET inhibitors, such as selpercatinib and pralsetinib, are currently under investigation [139,140]. In 2020, a phase 1–2 clinical trial enrolled patients with thyroid cancer harboring an activating RET alteration for treatment with selpercatinib (LIBRETTO-001). In this trial, one patient with ATC and 3 with PDTC were enrolled. Interestingly, the patient with ATC reached PR as best response as well as 2 out of 3 patients with PDTC, while the other one with PDTC obtained SD [139]. Furthermore, selpercatinib presents a more tolerable toxicity profile with a rate of adverse events ≥ III of only 30% compared to other targeted therapies and the most common reported adverse events were hypertension, increased alanine or aspartate aminotransferase level, hyponatremia and diarrhea. Likewise, Cabanillas et al. have recently presented data about the use of larotrectinib, a NTRK fusion gene inhibitor, in 7 patients with ATC. Intriguingly, 3 out of 7 reached PR and SD, while 3 patients experienced PD [141]. Grade ≥ III adverse events occurred in 46% of patients, although only 7% of patients presented ones that were considered related to larotrectinib [141]. Recently, an excellent response was documented with crizotinib in one patient with ATC, harboring ALK-RET fusion gene [142].

Other agents such as HDAC inhibitors have been used but with disappointing results (NCT03002623 trial).

### 4.3. Immunotherapy

Immunotherapy is inducing a deep change in anticancer therapy, regulating immune cells attack against neoplastic cells. Interestingly, many reports showed that ATC presents higher PD-L1^+^ cells compared to DTC, proposing PD/PD-L1 pathway as targetable [101,143], and, as shown above, preclinical data produced interesting result in mouse model [108]. Accordingly, PD-1 antibodies (e.g., pembrolizumab and spartalizumab), after promising data about their use in BRAF-mutated melanoma [144], have been used as single agents in patients affected by ATC [145].

Pembrolizumab induced a durable response (16 months) in one patient with unresectable ATC: after its second cycle the patient referred a significant improvement of dysphagia and after 3 cycle a complete response was almost reached [146]. However, after a severe toxicity related to pembrolizumab (grade 4 colitis), it was suspended and the patient died 8 months later, after the appearance of cerebral metastasis [146]. On the other hand, it seemed to do not produce the same result when co-administrated with chemoradiotherapy. Chintakuntlawar et al. treated 3 patients with pembrolizumab and chemoradiotherapy, but, in spite of a prompt an early tumor response, all patients passed away <6 months [147].

Spartalizumab toxicity and efficacy were evaluated in a phase I/II trial enrolling 42 patients with locally advanced and/or metastatic anaplastic thyroid carcinoma [148]. The overall response rate was 19% in the whole cohort, while it was higher in patients defined as PD-L1–positive (29%), and even better in the subset of patients with PD-L1 expression > 50% (35%). In this last subset of patients, the 1-year survival rate reached 52.1% [148]. About toxicity profile, the most frequent adverse events were diarrhea, pruritus, fatigue, and pyrexia and grade ≥ III adverse events related to treatment were observed in 10% of patients [148].

### 4.4. Multimodal Therapy

Considering the promising data about single regimens, many clinicians proposed a multimodal therapy against ATC in order to reduce therapy resistance. Moreover, driver mutations such as BRAF were proposed as master regulators of immune TME in thyroid cancer [114,149]. Multimodal therapy against immune TME and main mutated pathways could induce a deep inhibition of ATC growth and progression, evading resistance mechanisms.

In 2018, Cabanillas et al. showed one case of locally aggressive unresectable ATC treated with neo-adjuvant therapy composed by dabrafenib, trametinib and pembrolizumab (DTP) [150]. Interestingly, the patient had a relevant response, allowing a complete surgical resection followed by postoperative chemoradiation. Likewise, other 4 clinical cases have recently been reported about DTP use as neoadjuvant therapy in ATC, with unexpected high PFS (19.5, 95% CI: 13.75–24.5, months) [151].

Immunotherapy has been proposed also in adjuvant therapy with dabrafenib and trametinib or lenvatinib. Iyer et al. [152] used pembrolizumab as salvage therapy in 5 patients treated with dabrafenib and trametinib, 1 with trametinib, and 6 with lenvatinib. Although 2 patients experienced PD, 5 patients had PR and 5 had SD and, from the start of targeted therapies, the median OS was 10.4 months (95% CI = 6.02, 14.83, range 5.4–40 months) [152]. In this series, fatigue, anemia and hypertension were the most common AEs associated with this combination and drug-induced rash and altered mental status (likely related to PD) induced drug interruption [152]. Similarly, Dierks et al. showed interesting results about lenvatinib and pembrolizumab combined treatment both in ATC and PDTC: 5/6 patients with ATC reached CR/PR and 2/2 with PDTC obtained PR [153]. Similarly, nivolumab (anti-PD1 antibody) was added to vemurafenib in patients affected by metastatic ATC, obtaining a prolonged response (more than 20 months) [154]. According to these results, new clinical trials are ongoing (e.g., NCT03181100).

In 2017, 6 patients with PDTC and 2 with ATC were enrolled to receive sorafenib and temsirolimus (mTOR inhibitor) in a non-randomized clinical trial. In one hand, results in patients with PDTC were encouraging and 4 patients reached PR and 2 SD; on the other hand, one patient with ATC had PR and the other one had PD [155]. Furthermore, 14% of enrolled patients discontinued the treatment for toxicity and most common adverse events grade ≥ 3 were hyperglycemia, fatigue, anemia, and oral mucositis [155].

## 5. Conclusions

PDTC and ATC are rare but, unfortunately, they are lethal although a relevant different 5-year survival rate (5 years vs. 6 months). Nowadays, we know many elements of their genetic landscape and tumor microenvironment. This knowledge helped the scientific community to identify therapies which specifically target these cancers. Some of them (e.g., DTP) has recently reached the clinical practice and could be prescribed for BRAF^V600E^ mutated ATC. However, their therapeutic benefit is still scarce and many other studies are necessary to answer these unmet clinical needs.

## Figures and Tables

**Table 1:** ATC and PDTC genetic landscape: somatic mutations.

Cellular Function		Gene	Mutation Rate (%)	
			ATC [21,29]	PDTC [18,22,23,24]
Intracellular signaling	MAPK pathway	*BRAF*	27.63	15.38–33.33
		*NRAS*	19.25	4.35–30.77
		*NF1*	5.56	0
		*KRAS*	4.92	0–5.31
		*HRAS*	4.51	2.45–4.88
	PI3K-AKT pathway	*PIK3CA*	11.24	2.38–19.51
		*PTEN*	9.27	0–4.35
		*NF2*	5.10	0
		*IRS1*	3.64	-
		*AKT1*	-	0-8.70
	WNT pathway	*AXIN1*	4.51	-
		*CTNNB1*	3.88	0–2.44
		*APC*	3.05	17.39
Cell cycle regulation		*TERT promoter*	75	21.95–40.48
		*TP53*	45.67	8.33–43.48
		*ATM*	4.91	7.14–13.04
		*RB1*	4.36	1.19–4.35
		*CDKN2A*	4.01	-
Chromatin remolding		*KMT2D*	4.42	-
		*CREBBP*	4.17	-
		*ARID2*	3.93	-
		*ARID1A*	3.69	-
		*DNMT3A*	3.38	-
		*KMT2A*	3.36	-
DNA damage response		*MDC1*	3.18	-
		*MSH2*	3.05	-
Protein metabolism		*EIF1AX*	9.24	4.88–10.71
		*CALR*	4.85	-
		*RBM10*	3.38	-

**Table 2:** ATC and PDTC genetic landscape: gene fusions.

Gene Fusions		Mutation Rate [16,18,29,44,46]	
		ATC	PDTC
PAX8-PPARγ Fusions		0	3/84
NTRK fusions	*NTRK1-IRF2BP2*	1/126	0
	*NTRK2-CRNDE*	1/126	0
	*ETV6-NTRK3*	0	1/60
RET fusions	*CCDC6-RET*	2/126	3/84, 2/60
	*NCOA4-RET*	Case report	2/84, 1/23
	*PDCD10-RET*	0	1/60
	*TFG-RET*	0	1/60
ALK fusions	*STRN-ALK*	Case report	1/23
	*EML4-ALK*	0	2/84
BRAF fusions	*KIAA1549-BRAF*	Case report	0
	*SCRIB-BRAF*	0	1/60
Other fusions	NUT-BRD4	1/33	0
